# The Significance of the Heterogeneity of Cancer-Associated Fibroblasts in Tumor Microenvironments

**DOI:** 10.3390/metabo16020120

**Published:** 2026-02-09

**Authors:** Daiki Imanishi, Hinano Nishikubo, Dongheng Ma, Hongdong Gao, Tomoya Sano, Canfeng Fan, Takashi Sakuma, Yurie Yamamoto, Masakazu Yashiro

**Affiliations:** 1 Department of Molecular Oncology and Therapeutics, Graduate School of Medicine, Osaka Metropolitan University, 1-4-3 Asahi-machi, Abeno-ku, Osaka 545-8585, Japan; sy23003h@st.omu.ac.jp (D.I.); sn23089k@st.omu.ac.jp (H.N.); sg24231c@st.omu.ac.jp (D.M.); gaohomgdong99@gmail.com (H.G.); sb24524y@st.omu.ac.jp (T.S.); y23703o@omu.ac.jp (C.F.); j22519d@omu.ac.jp (T.S.); f21269a@omu.ac.jp (Y.Y.); 2 Cancer Center for Translational Research, Graduate School of Medicine, Osaka Metropolitan University, 1-4-3 Asahi-machi, Abeno-ku, Osaka 545-8585, Japan

**Keywords:** tumor microenvironment, tumor heterogeneity, cancer-associated fibroblasts, cell to cell interaction, precision medicine, chemoresistance

## Abstract

The tumor heterogeneity that is frequently observed in cancer tissues comprises not only cancer cells but also stromal cells in the tumor microenvironment. One of the major components of tumor stroma, i.e., cancer-associated fibroblasts (CAFs), play crucial roles in tumor progression and the tumor response to chemotherapy. The known subtypes of CAFs are antigen-presenting CAFs (apCAFs), myofibroblastic CAFs (myCAFs), and inflammatory CAFs (iCAFs). It has been speculated that (i) the heterogeneity of CAF subtypes might contribute to tumor progression; (ii) cell-to-cell interactions among CAF subtypes in tumors might be associated with the development of various types of carcinomas, and (iii) juxtracrine and/or paracrine signaling from CAFs may play important roles in this development. A clarification of the mechanisms that underlie the tumoral heterogeneity of CAFs could contribute to cancer treatment as precision medicine. This review explains the significance of CAF heterogeneity in tumor microenvironments, especially concerning the CAF subtypes.

## 1. Introduction

Tumor heterogeneity refers to the presence of multiple clones with biological heterogeneity of not only cancer cells but also stromal cells in a tumor microenvironment. Cancer-associated fibroblasts (CAFs) play a crucial role in tumor progression, and the heterogeneity of CAFs reflects their diverse functions within tumor microenvironments, as CAFs contribute to various tumorigenic processes. These fibroblasts exhibit their heterogeneity as several subtypes that have been reported to be associated with tumor heterogeneity [1]. Several studies have established that CAFs remodel the extracellular matrix (ECM) through interactions with cancer cells, facilitate cancer invasion, modulate the immune environment surrounding a tumor, and promote cancer metastasis [2,3]. It has also been suspected that paracrine signaling from CAFs might play important roles in tumor progression. A precise clarification of CAF heterogeneity could contribute to cancer treatment as precision medicine. Here, we review the existing research concerning the significance of tumor heterogeneity, particularly concerning the subtypes of CAFs.

## 2. CAF Heterogeneity in Tumor Microenvironments

Three distinct main subtypes of CAFs are known: a subtype that expresses major histocompatibility complex (MHC) class II and CD74, called ‘antigen-presenting CAFs (apCAFs)’ [4]; a subtype that expresses alpha smooth muscle actin (α-SMA), called ‘myofibroblastic CAFs (myCAFs); and a subtype that actively responds to transforming growth factor beta (TGF-β), expresses a low level of α-SMA, and secretes the inflammatory mediator interleukin (IL-6), called ‘inflammatory CAFs (iCAFs)’ [5]. apCAFs differ from macrophages and dendritic cells, presenting antigen via MHC class II expression following antigen uptake [6,7]. It has been demonstrated that iCAFs contribute to chemoresistance in various types of cancers, including pancreatic cancer, breast cancer, and colorectal cancer [8].

Diverse immune microenvironments exhibiting different responses to the same treatment regimen have been identified in different metastatic sites of the same ovarian cancer patient [9]. A similar scenario is presumed for CAFs, which may influence the nature of immune responses and treatment reactivity across different sites. Indeed, differences in fibroblast characteristics have been observed between micrometastases and major metastases [10]. Furthermore, fibroblasts at metastatic sites differ from those in the primary tumor [11,12]. This supports the existence of functionally and phenotypically distinct CAF populations.

The development of CAF heterogeneity is frequently affected by factors in tumor microenvironments [13]. The above-described CAF subtypes are characterized by distinct histological features and transcriptome profiles. Liu et al. revealed that the spatial organization of CAFs forms distinct cellular neighborhoods within the tumor microenvironment, creating microenvironments with unique properties that influence the transcriptional state and functional roles of CAFs [14]. The CAF subtypes also exhibit distinct morphological features. myCAFs are larger than typical fibroblasts, exhibiting a spindle shape or stellate shape, and are considered to have a morphology similar to smooth muscle cells. iCAFs and apCAFs have fewer specific descriptions regarding morphological characteristics and are primarily defined by their functional and secretory properties [15]. The spatial CAF subtypes have been observed to vary among tumor histologic types and clinical stages and to correlate with patient prognoses. A more thorough understanding of CAF heterogeneity is desired, as this could aid in the development of targeted therapies against carcinomas in the near future.

## 3. Interactions Between CAFs and Cancer Cells in Tumor Microenvironments

### 3.1. The Effects of Paracrine Signaling from CAFs

**Figure 1:**
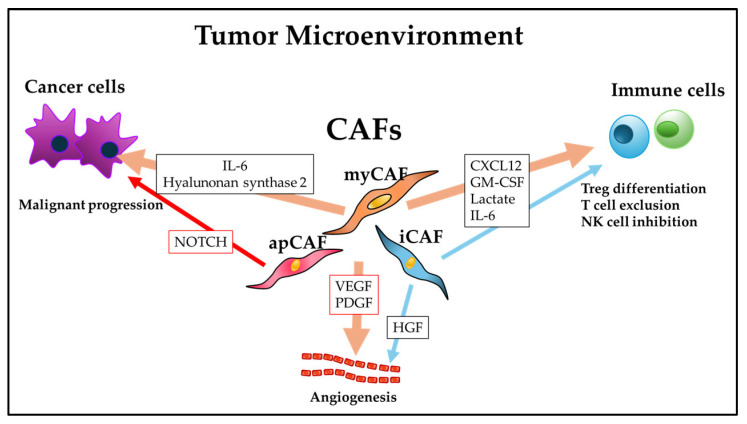
The effects of cancer-associated fibroblasts (CAFs) on cancer cells and stromal cells in the tumor microenvironment.

CD146+ CAFs promote the proliferation and stemness of malignant cells via an IL-6/IL-6R axis [16]. Other markers for cancer-promoting CAFs include podoplanin among iCAF markers and CD74+ among apCAF markers. IL-6 from human primary CAFs was shown to activate STAT3 signaling, which stimulates the epithelial–mesenchymal transition (EMT) and the proliferation, migration, and invasion of cancer cells [17]. Although multiple preclinical studies of the significance of CAFs and these cells’ roles have been conducted for a broad range of cancer types, the treatment strategies examined in those studies often failed to demonstrate a clinical benefit, and it was proposed that these difficulties regarding clinical applications of CAFs may be due at least in part to the heterogeneity of the CAF subtypes [18]. The schematic provided as Figure 1 depicts the effects of CAFs on cancer cells [19,20] and immune cells [19,21] in tumor microenvironments. Each CAF shows different aspects in a tumor, suggesting the CAF heterogeneity in tumor microenvironments.

Paracrine signaling from CAFs to malignant cells has been documented in various types of cancer. The CAF-S4 subtype, which is characterized by increased expression of muscle contraction and actin cytoskeleton, promotes the ability of cancer cells to invade tissues [22]. CAF-S1 myofibroblasts secrete C-X-C motif chemokine 12 (CXCL12, which is also known as stromal cell-derived factor-1 [SDF-1]) to mobilize CD4+ T cells and CD25+ T cells, stimulating their differentiation into regulatory T cells [23]. Paracrine signaling from CAFs modulates the body’s innate immune system. CAFs that are positive for neuroendocrine tumor (NET) G1 directly suppress the functioning of natural killer cells by secreting granulocyte-macrophage colony-stimulating factor (GM-CSF), IL-1β, IL-6, and IL-8 [19]. TGF-β-driven CAFs that are positive for the protein known as ‘leucine-rich repeat containing (LRRC)15’ were observed to suppress the activity of tumor-infiltrating CD8+ T cells by suppressing the expressions of tumor necrosis factor-alpha (TNF-α) and interferon-gamma (IFN-γ) [24].

In contrast to the extensive evidence for paracrine signaling from CAFs to malignant cells, evidence for paracrine signaling from CAFs to malignant cells is limited. Breast cancer exemplifies this; in the CAF-S4 subtype, characterized by enhanced expression of genes related to muscle contraction and actin cytoskeleton regulation, highly expressed NOTCH receptors have been reported to underpin its contractility and ability to promote malignant cell invasion [22]. Another example is hepatocellular carcinoma (HCC), where patient-derived αSMA-positive CAFs have been shown to promote the regeneration and proliferation of liver cancer stem cell-like cells by activating Notch3 signaling in both in vitro co-culture and subcutaneous co-transplant mouse models [25].

Interactions between fibroblasts and immune cells also occur in the metastatic tumor microenvironment (TME). In pancreatic ductal adenocarcinoma (PDAC) liver metastases, myoblast-like metastasis-associated fibroblasts (myMAFs) secrete osteopontin, suppressing macrophage-mediated CD8+ T cell infiltration and activation [26]. Furthermore, in lung metastasis-associated fibroblasts from breast cancer, IL-33 is upregulated compared to normal lung fibroblasts, promoting the mobilization of neutrophils, eosinophils, and T cells. This mediates type 2 immunity, forming a metastasis-supporting niche [27]. Moreover, in breast cancer lung metastasis, cyclooxygenase-2 (COX-2)-positive MAFs induce dendritic cells and monocytes toward an immunosuppressive phenotype via prostaglandin E2 signaling, ultimately suppressing T cell proliferation and NK cell cytotoxicity [28].

Furthermore, in malignant melanoma and colorectal cancer cells, CAFs induce high PD-L1 expression and activation of PI3K/AKT signaling, leading to the disappearance of T cells in the antitumor immune response [29]. Furthermore, CAFs can inhibit antitumor immune responses by suppressing dendritic cells, which are essential for T lymphocyte activation. For example, it has been revealed that CAFs secrete WNT2 in esophageal squamous cell carcinoma and colorectal cancer. WNT2 suppresses the role of dendritic cells in antitumor T cell responses via the SOCS3/p-JAK/p-STAT signaling pathway [30]. Furthermore, CAFs have been shown to reduce immune efficacy by mobilizing granulocytes and monocytes and suppressing dendritic cell function [31,32]. Increased IL-33 expression in metastasis-associated fibroblasts stimulates type 2 immunity, mediating the mobilization of eosinophils, neutrophils, and inflammatory monocytes, and affects the function of these immune cells within the tumor microenvironment [27].

### 3.2. ECM Remodeling

CAF metabolism directly impacts ECM organization. Massive collagen production by activated fibroblasts requires increased proline synthesis from circulating glutamine, which depends on elevated pyrroline-5-carboxylate reductase (PYCR1) expression in CAFs. This expression is epigenetically regulated by histone acetyltransferase EP300 and acetyl-CoA levels [33].

CAFs have been shown to produce tenascin C and fibrillar collagen, maintain tumor stiffness, and enhance focal adhesion kinase (FAK)-dependent tumor-promoting signaling in pancreatic cancer [34]. Tumor growth is promoted by hyaluronan synthase 2 expressed by myCAFs, rather than type I collagen, as demonstrated in conditional gene knockout mouse models of intrahepatic cholangiocarcinoma [20]. The use of single-cell RNA sequencing in an investigation of non-small cell lung cancer revealed that myosin heavy chain (MYH)11+ αSMA-positive CAFs and fibroblast activation protein (FAP)-positive αSMA+ CAFs produce different collagen types, and that both of these CAF subtypes independently reorganize collagen into dense, aligned fibers, limiting contact between malignant cells and T cells and promoting the exclusion of T cells [35].

### 3.3. Metabolite Exchange

During tumor progression, CAFs frequently undergo a metabolic switch to aerobic glycolysis (Reverse Warburg Effect [36]), resulting in the secretion of energy-rich metabolites. These are taken up by cancer cells, serving as fuel to promote their anabolic metabolism [37,38,39]. Maria Apicella [40] demonstrated that cancer cells undergo a metabolic switch during MET or EGFR TKI therapy, increasing lactate production, which in turn supports CAFs in producing resistance-promoting growth factors. In the same resistant tumors, this metabolic switch occurred not only in cancer cells but also in CAFs, which exhibited enhanced glycolytic metabolism. This Reverse Warburg Effect allowed CAFs to maintain hepatocyte growth factor (HGF) overexpression indefinitely even in environments devoid of cancer cells [40].

Lipid-rich ATP-binding cassette (ABC)A8a+ CAFs were reported to supply lipids to malignant cells as a substrate for mitochondrial oxidative phosphorylation [41]. During tumorigenesis, CAFs secrete abundant lipids, promoting malignant cell proliferation via a lysophosphatidic acid-autotaxin (LPA-AKT) axis [42]. CAFs also supply alanine to malignant cells through autophagy-dependent secretion and the solute carrier family 1 member 4 (SLC1A4) transporter system [37,43]. A CAF subtype that is active in pancreatic cancer was shown to rely on glycolysis to supply metabolic intermediates to malignant cells, which depend on mitochondrial oxidative phosphorylation [44]. CAF-derived metabolites have long been recognized as key factors promoting immunosuppression [45]. Glycolytic CAFs release lactate, which affects the differentiation of CD4+ T-cells, thereby reducing the numbers of antitumor type 1 helper T cells and promoting an increase in the numbers of regulatory T cells [21].

### 3.4. Interactions Between CAFs and Perivascular Cells

CAF subtypes regulate tumor angiogenesis via paracrine and apocrine signaling. While previous studies demonstrated that CAFs secrete multiple proangiogenic factors such as vascular endothelial growth factor (VEGF), platelet-derived growth factor (PDGF), and HGF [46], recent research is increasingly revealing associations between tumor angiogenesis and specific CAF subsets. In multiple cancers, the adhesion protein CD146 identifies “vascular CAFs” primarily localized in perivascular areas and expressing vascular development-related genes [16,47,48,49]. In endometrial cancer, CD146+ CAFs interact with malignant cells via IL-10/JAK1/STAT3 signaling, inducing vascular mimicry [49]. As another example, abnormal KRAS signaling in CRC malignant cells activates the transcription factor CP2 (TFCP2) within CAFs, converting them to a lipid-rich, VEGF-A-secreting phenotype [50]. This further promotes angiogenesis [50]. Importantly, lipid-rich CAFs and the TFCP2 signature correlate with poor prognosis in CRC patients, while targeting TFCP2 improves survival in CRC mouse models [50]. Hypoxic breast cancer CAFs also secrete VEGF-A and induce angiogenesis [51]. Furthermore, in multiple myeloma, αSMA-positive and FAP-positive patient-derived CAFs secrete exosomes containing microRNA-21, which promotes angiogenesis upon uptake by endothelial cells (ECs) [52].

In gastric cancer, following cisplatin and paclitaxel treatment, the USP7/hnRNPA1 axis is activated in CAFs, leading to miR-522 expression. This inhibits ALOX15, reduces lipid-ROS accumulation within cancer cells, and ultimately results in decreased chemotherapy sensitivity [53]. CAFs also secrete IL-8, which may activate the NF-κB signaling pathway in gastric cancer and mediate chemotherapy resistance [54].

## 4. The Roles of CAFs in Resistance to Cancer Therapies

CAFs also have an impact on tumors’ resistance to chemotherapy. In ovarian cancer, fibroblasts interact with other cell types through various pathways, ultimately contributing to higher chemotherapy resistance [55]. In studies of bladder and pancreatic cancer, CXCL12+ CAFs increased the expression of programmed cell death ligand 1 (PDL1) and promoted the cancer cells’ migration and invasion [56,57]. The results of an investigation of oral squamous cell carcinoma demonstrated that CXCL12+ CAFs recruit monocytes through a CXCL12–CXCR4 (CXD motif chemokine receptor 4) pathway and induce their differentiation into M2 macrophages, which resulted in the promotion of tumor cell proliferation and a reduction in apoptosis in this cancer type [58]. It was also observed that CAFs promote the survival and migration of mycosis fungoides cells by secreting CXCL12.

Together, the above-cited findings suggest that targeting the CXCL12–CXCR4 axis may change the tumor microenvironment and thus enhance the efficacy of anticancer therapies [59]. CXCL12 from CAFs modulated cancer stem cell phenotypes and promoted chemoresistance in multiple tumors via an interaction between CXCR4 and Wnt/β-catenin pathways in cancer cells [60]. In their analysis of the evolutionary pathways of tumor cells and the tumor microenvironment in ovarian cancer, Wang et al. revealed that CXCL12-expressing fibroblasts influenced patient prognoses and treatment outcomes through spatial proximity interactions with tumor cells, providing a novel perspective for the prevention and treatment of ovarian cancer [61].

## 5. Targeted Therapy Against CAFs

Two major immunotherapy strategies targeting CAFs are being explored: (1) the direct elimination of CAFs by targeting surface markers such as fibroblast activation protein (FAP), and (2) the suppression of CAFs’ functions by modulating a key signaling molecule, i.e., TGF-β. Although treatments designed to inhibit CAFs have demonstrated some efficacy in preclinical animal models, the success of these treatments in clinical trials has been limited [62]. Some emerging therapies targeting CAF-associated signaling pathways (e.g., TGF-β inhibitors and IL-6 blockade) have demonstrated the ability to modulate both CAF activity and inflammatory responses [63,64]. Combining TGF-β inhibitors with the chemotherapy drug gemcitabine and anti-PD-L1 antibodies has been shown to yield superior antitumor effects [65,66,67]. Tocilizumab, an IL-6/JAK/STAT3 inhibitor, has demonstrated the potential to enhance immune responses and improve tumor control [68]. These findings suggest that targeting CAFs may improve the efficacy of cancer therapy.

CAFs are known to contribute to tumor progression [69] and chemotherapy resistance [70], which is thought to be at least partially attributable to the secretion of TGF-β ligands by CAFs [71]. TGF-β not only induces tumor cell plasticity and cancer cell heterogeneity and promotes cancer stemness via EMT, but it also significantly impacts the tumor microenvironment. CAFs significantly contribute to the production of ECM proteins within the tumor microenvironment [72], and this contribution can be stimulated by TGF-β ligands. High ECM production creates physical and chemical barriers to immune-cell infiltration [18,73] and generates physical tension within the tumor, correlating with enhanced TGF-β signaling and chemotherapy resistance, as observed in gastric cancer [74]. Studies using low-molecular-weight inhibitors targeting TGF-β receptor type 1 (TGF-βR1) have demonstrated reduced tumor angiogenesis, enhanced vascular stability, and decreased interstitial tumor pressure in breast cancer, skin squamous cell carcinoma, hepatocellular carcinoma, and glioblastoma as responses to an inhibition of TGF-β signaling [75,76]. TGF-β is also a key regulator of fibroblast-to-CAF transformation and influences not only tumor cells [69,77] but also the mobilization and cellular functions of diverse types of immune cells within tumors [18,69,73,78].

In 2020, Dominguez et al. discovered that TGF-β stimulates a major subset of CAFs in pancreatic cancer, which were identified by the expression of LRRC15 [79]. They observed that in clinical trials of immunotherapy across various cancer types, a high TGF-β CAF signature correlated with poor patient survival [79]. Subsequent work by the same group revealed that LRRC15^+^ CAFs promoted tumor progression and resistance to anti-PD-L1 therapy by suppressing effector T-cell function in various mouse tumor models [24]. CAFs have also been reported to modulate the tumor-associated immune environment (especially the expression level of PD-1/PD-L1) via various cytokines, including IL-6 [23,80]. Building on the research regarding CAFs and pancreatic cancer immunotherapy resistance, it was observed that esophageal cancer CAFs may also contribute to immunotherapy resistance by upregulating the expression of PD-L1 [81].

Furthermore, one promising therapeutic approach involves “normalizing” the CAF. Targeting the vitamin D receptor in pancreatic cancer is one such example. Treatment with vitamin D receptor ligands reverted activated astrocytes to a quiescent state, reducing the disease’s invasiveness [82,83]. Therapies aimed at normalizing the CAF in this manner are also gaining attention.

## 6. Conclusions

The clarification of the significance of CAF heterogeneity in tumor microenvironments remains incomplete. Although the effects of CAF subtypes on the progression of cancer have not been established, continued research to determine the precise functional mechanisms that underlie the heterogeneity of CAFs could be a crucial key for cancer treatment in the future.

## 7. Emerging Concepts and Perspectives

Recently, immunotherapy has taken the forefront in cancer treatment. While the role of CAFs in immunotherapy is attractive, the immune function of each CAF subtype remains unclear. Among the three CAF subtypes, apCAF is the least known. One known function of apCAF is effective against the tumor immune system by activating CD4+ cells. Ren Y. et al. [84] reported that apCAFs engaged in crosstalk with M2-macrophages via TGF-β signaling pathways. The co-localization of apCAFs and M2 macrophages at the tumor periphery indicated the formation of an immunosuppressive niche, suggesting the targeting of apCAF-M2 macrophage interactions to overcome immune resistance in glioma. Zhou, Z. et al. [85] reported that pCAF subtypes rich in immune elements of activation of genomic instability pathways are associated with poor prognosis of bladder cancer; they might show promising clinical response to immunotherapy. Understanding heterogeneity of CAF subtypes might provide a basis for diagnosis and screening of bladder cancer. Chang, S. et al. [86] demonstrate that in situ CAF engineering is a promising strategy to remodel the tumor microenvironment and enhance immunotherapy in triple-negative breast cancer. These reports suggested that apCAF might be involved in immune responses among CAFs regardless of cancer type. Also, targeting the immune responses of apCAF could enhance the clinical efficacy of immunotherapy. Similarly, the development of drugs that remodel the distribution of apCAF and other subtypes of CAF in tumors might also be promising for the treatment of cancers. Furthermore, not only apCAF but also other subtypes could contribute to the immunotherapy for cancer. Analyzing the immune regulatory CAFs, especially apCAFs, might be necessary to predict sensitivity for cancer immunotherapy and maximize the benefits of tumor treatment in the near future.

## Data Availability

No new data were created or analyzed in this study.

## Abbreviations

The following abbreviations are used in this manuscript:

ABC	ATP-binding cassette
apCAF	antigen-presenting cancer-associated fibroblast
CAF	cancer-associated fibroblast
CXCL12	C-X-C motif chemokine 12
CXCR4	CXD motif chemokine receptor 4
ECM	extracellular matrix
EMT	epithelial–mesenchymal transition
FAK	focal adhesion kinase
FAP	fibroblast activation protein
GM-CSF	granulocyte-macrophage colony-stimulating factor
iCAF	inflammatory cancer-associated fibroblast
IFN-γ	interferon-gamma
IL	interleukin
LPA-AKT	lysophosphatidic acid-autotaxin
LRRC	leucine-rich repeat-containing
MHC	major histocompatibility complex
myCAF	myofibroblastic cancer-associated fibroblast
MYH	myosin heavy chain
NET G1	neuroendocrine tumor G1
PDL1	programmed cell death ligand 1
SLC1A4	solute carrier family 1 member 4
TGF-β	transforming growth factor beta
TGF-βR1	transforming growth factor beta receptor type 1
TNF-α	tumor necrosis factor-alpha
α-SMA	alpha smooth muscle actin
